# Clinical Assessment and Exercise Rehabilitation of Sarcopenia in Cardiovascular Disease

**DOI:** 10.3390/jcm15218637

**Published:** 2026-11-05

**Authors:** Soon Il Jeong, Un Hyo Baek, Hyun Seok Bang, Dae Yun Seo

**Affiliations:** 1 Department of Taekwondo, Dong-Eui University, Busan 47340, Republic of Korea; 65370@deu.ac.kr; 2 Department of Sports Science, Kyungnam University, Changwon 51767, Republic of Korea; unhyob@kyungnam.ac.kr; 3 Department of Sport Rehabilitation, College of Health, Social Welfare and Education, Tong Myong University, Busan 48520, Republic of Korea; 4 Department of Anatomy, College of Medicine, Inje University, Busan 47392, Republic of Korea

**Keywords:** sarcopenia, cardiovascular disease, heart failure, skeletal muscle metabolism, exercise training, cardiac rehabilitation

## Abstract

Sarcopenia complicates cardiovascular disease, but diagnostic definitions and outcome selection can substantially alter the interpretation of exercise rehabilitation. This critical review examines how these differences affect the translation of muscle biology into clinical assessment and treatment, with heart failure as the principal clinical setting. Targeted literature retrieval prioritized recent consensus statements, clinical studies, and intervention trials, while experimental evidence was considered separately. Human biopsy studies associate inflammatory and mitochondrial abnormalities with muscle dysfunction; causal manipulation of muscle-derived signaling remains predominantly experimental. European and Asian consensus frameworks differ in their thresholds and the diagnostic role of physical performance. These distinctions matter because trials frequently enroll patients with frailty or exercise intolerance without confirming sarcopenia. Resistance and combined interventions improve selected measures of strength or physical performance, whereas muscle quantity, diagnostic status, and clinical events may respond differently. A small coronary artery disease trial provides direct, short-term evidence in confirmed sarcopenia, but durability remains uncertain. Rehabilitation should therefore link an explicit diagnostic framework to individualized exercise, nutritional assessment, and repeated measurement of the intended outcomes. Future trials should establish whether improvements in defining muscle components persist and translate into sustained independence and fewer clinical events.

## 1. Introduction

Sarcopenia is a skeletal muscle disease for which reduced muscle mass, muscle strength, and muscle-specific strength constitute central conceptual components [1]. In cardiovascular disease (CVD), its assessment and management are complicated by ageing, physical inactivity, nutritional inadequacy, and disease-associated exercise intolerance [2]. Among older patients hospitalized for heart failure (HF), sarcopenia has been associated with increased mortality, supporting its prognostic relevance beyond the assessment of cardiac dysfunction alone [3]. Longitudinal community data also associate deterioration in sarcopenia status with incident CVD, although observational associations do not establish that reversing sarcopenia prevents cardiovascular events [4]. These findings support the evaluation of skeletal muscle impairment as a distinct component of cardiovascular assessment and rehabilitation [2,3,4].

The clinical burden is not restricted to symptomatic or advanced heart failure. In PAPRIKA-HF, sarcopenia defined using Asian Working Group for Sarcopenia (AWGS) 2019 criteria was associated with adverse outcomes in older outpatients with stage B heart failure [5,6]. Meta-analyses also associate multidimensionally defined sarcopenia with mortality and cardiovascular events, although diagnostic heterogeneity limits the transfer of pooled estimates to an individual population [7]. Comparisons across ejection-fraction phenotypes have produced heterogeneous prevalence estimates, with limited direct comparative prognostic evidence [8]. Accordingly, estimates of prevalence and prognostic significance should be interpreted within the context of the specific cardiovascular phenotype and the diagnostic criteria employed to define sarcopenia [8].

Exercise training is a core component of cardiovascular rehabilitation, with aerobic and resistance exercise addressing complementary aspects of exercise capacity and muscular function [9]. Human biopsy studies in heart failure with preserved ejection fraction (HFpEF) have identified altered inflammatory gene expression and reduced mitochondrial respiratory capacity [10,11]. Experimental exercise studies and a human training substudy indicate that skeletal muscle metabolic pathways remain responsive to intervention, although the magnitude and persistence of adaptation vary by model and training modality [12,13]. Clinical trials have reported differing responses across muscle strength, muscle quantity, and physical performance, emphasizing the need for outcome-specific interpretation [14,15]. Increased strength or physical performance does not, by itself, demonstrate increased muscle mass or resolution of sarcopenia [14,15,16,17].

The European Association of Preventive Cardiology (EAPC) scientific statement synthesizes exercise and nutritional approaches to CVD-associated sarcopenia and acknowledges the limited intervention evidence in patients with confirmed sarcopenia [2]. Building on this work, the present critical review focuses on the relationship between diagnostic classification, participant eligibility, and the interpretation of rehabilitation outcomes. The Global Leadership Initiative in Sarcopenia (GLIS) provides a conceptual definition [1], whereas the revised European Working Group on Sarcopenia in Older People consensus (EWGSOP2) and successive AWGS frameworks provide operational criteria with differing roles for muscle strength, muscle quantity, and physical performance [6,16,17].

With heart failure as the principal clinical setting, we distinguish evidence in patients with diagnostically confirmed sarcopenia from evidence in broader cardiovascular populations selected for frailty, low strength, or exercise intolerance. We consider muscle strength, muscle quantity, physical performance, exercise capacity, and clinical events as distinct outcomes. The specific contribution of this review is to connect these population and outcome distinctions with clinical assessment and rehabilitation, clarifying which findings directly support the management of confirmed sarcopenia and which provide indirect guidance. In particular, functional improvement is distinguished from a documented change in sarcopenia status or a reduction in clinical events.

Sarcopenia should also be distinguished from frailty, malnutrition, and cachexia, which frequently coexist but describe different clinical constructs. Sarcopenic obesity requires assessment of excess adiposity together with reduced muscle mass and function; body mass index alone cannot identify this phenotype [18,19]. A prospective heart failure cohort associated both sarcopenia and sarcopenic obesity with adverse outcomes, while a cohort of older adults with CVD reported particularly unfavorable outcomes when sarcopenia coexisted with visceral obesity [20,21]. These findings support direct assessment of body composition and function when obesity might otherwise obscure muscle impairment [18,21].

### Scope and Literature Selection

This focused critical review examines how diagnostic definitions, study populations, and outcome selection influence the interpretation of exercise rehabilitation for sarcopenia in cardiovascular disease, with heart failure as the principal clinical setting. During revision, a targeted PubMed update was performed on 27 October 2026 for English-language publications dated 1 January 2021 to 27 October 2026. Five searches addressed diagnostic frameworks, clinical assessment, exercise rehabilitation, HFpEF muscle mechanisms, and nutrition or weight management. Titles were inspected for relevance, and selected available abstracts were examined to clarify study design, population, and outcomes. The complete search strategies are provided in Supplementary Table S1. This update supplemented the literature already cited; earlier foundational consensus documents and pivotal trials were retained where relevant.

Evidence was prioritized according to its contribution to the diagnostic, mechanistic, or rehabilitation questions addressed in the review. Original consensus documents informed the comparison of diagnostic frameworks. Cardiovascular studies involving participants who met an explicit sarcopenia definition were distinguished from studies enrolling broader cardiovascular populations or participants selected for frailty, low strength, or impaired physical performance. Relevant findings from other older populations were considered indirect evidence. Human mechanistic observations and experimental interventions were interpreted separately.

Intervention findings were considered according to the outcomes measured, and publications from shared trials or cohorts were treated as related evidence rather than independent replication. Reports without relevant diagnostic, mechanistic, or rehabilitation findings were not prioritized. Protocols without intervention results were not used to establish treatment efficacy. Retrieval and relevance assessment were targeted and non-exhaustive; no formal systematic risk-of-bias assessment, certainty grading, or meta-analysis was undertaken.

Key reports were appraised qualitatively in relation to the specific inference under consideration. Potential sources of bias were considered in relation to participant selection, allocation, outcome assessment, and attrition. For intervention studies, appraisal also considered sample size, comparator selection, prespecified outcomes, precision of effect estimates, and follow-up duration. Between-group effects were distinguished from within-group changes, and confidence intervals were considered alongside clinically meaningful thresholds when available. Small pilot samples were treated as limitations in precision rather than automatically indicating high risk of bias. Diagnostic investigations were considered in relation to their reference definitions, case prevalence, and validation populations. These considerations guided the interpretation of findings without assigning numerical quality scores or formal certainty grades.

## 2. Skeletal Muscle Alterations in Cardiovascular Disease

Skeletal muscle impairment in CVD encompasses reduced muscle quantity, impaired force generation, and diminished exercise capacity, with contributions from physical inactivity, nutritional deficits, ageing, and disease-associated metabolic disturbances [2]. These manifestations should not be treated as interchangeable indicators of a single biological abnormality [1]. Human and experimental studies provide complementary evidence concerning skeletal muscle inflammatory and metabolic abnormalities [10,11]. Their relevance to clinical sarcopenia depends on whether the investigated population also meets a defined diagnostic framework and whether the molecular findings relate to directly measured muscle or functional outcomes [1,2].

Clinical observations support the coexistence of cardiac and muscular limitations without establishing that either fully explains the other. Mechanistic reviews describe interacting inflammatory, metabolic, and proteostatic processes rather than a single pathway sufficient to explain all cases [22]. This heterogeneity is also evident experimentally: metabolic and hypertension-driven HFpEF rat models differed in muscle atrophy, force generation, and titin phosphorylation [23].

### 2.1. Inflammation and Muscle Impairment

Human muscle studies have identified inflammatory abnormalities in heart failure, but their observational designs limit causal interpretation [10]. Saw et al. reported increased expression of selected inflammatory transcripts in vastus lateralis biopsies from patients with HFpEF; myofiber atrophy, fibrosis, and reduced capillary density were demonstrated in the accompanying mouse model [10]. These findings should be interpreted separately because the structural abnormalities observed experimentally were not uniformly demonstrated in the human samples [10].

Sex-dependent differences in skeletal muscle immune cell composition and associations with muscle mass and strength have also been reported in heart failure with reduced ejection fraction (HFrEF), indicating heterogeneity in the inflammatory phenotype [24].

Exploratory studies have identified exercise-related peripheral blood transcriptomic responses and changes in inflammatory markers during cardiac rehabilitation [25,26]. These observations characterize systemic responses to exercise but do not establish that suppression of a specific inflammatory pathway mediates muscle recovery or reverses sarcopenia [25,26]. Accordingly, these molecular and circulating measures should be interpreted alongside direct assessments of muscle quantity, strength, and physical performance.

### 2.2. Mitochondrial Metabolism

Human studies support an association between impaired skeletal muscle oxidative metabolism and exercise intolerance in HFpEF. In a cross-sectional comparison of 27 older patients with HFpEF and 45 age-matched controls, Scandalis et al. identified lower mitochondrial respiratory capacity associated with poorer exercise capacity and physical performance [11]. Potential confounding by adiposity, comorbidity, and unmeasured habitual activity limits attribution of these findings specifically to HFpEF [11]. Complementary multimodality assessment identified abnormalities in muscle oxidative metabolism and fiber composition [27]. These findings characterize an oxidative impairment phenotype without establishing that mitochondrial dysfunction independently causes sarcopenia.

The OptimEx muscle biopsy substudy provides evidence of exercise-associated molecular adaptation. Changes in energy metabolism and satellite cell measures were more evident after supervised high-intensity interval training than after moderate continuous training, despite functional improvement with both modalities; these molecular effects were not maintained at 12 months. This divergence cautions against assuming that a measured molecular change necessarily mediates functional improvement [13].

Experimental studies further investigate exercise-responsive substrate metabolism and the role of skeletal muscle iron regulation in oxidative energy production and exercise tolerance [12,28]. These findings identify candidate mechanisms for human investigation. Clinically, oxidative measurements should be interpreted alongside muscle strength and exercise testing because exercise intolerance also reflects cardiac, pulmonary, vascular, and autonomic limitations [29,30].

### 2.3. Proteostasis and Muscle-Derived Signaling

Evidence concerning muscle-derived signaling combines observational human findings with experimental tests of selected mechanisms. Jheng et al. investigated a pathway linking skeletal muscle sirtuin 3 (SIRT3) deficiency, lysyl oxidase-like 2 (LOXL2), and pulmonary vascular remodeling in HFpEF-associated pulmonary hypertension [31]. Human samples supported an association between this pathway and the disease phenotype, whereas muscle-specific Loxl2 deletion provided evidence of a mechanistic contribution in mice [31]. The experimental findings therefore establish a role within the investigated models rather than demonstrating therapeutic efficacy in patients.

Similarly, increased musclin expression and reduced peroxisome proliferator-activated receptor-γ coactivator-1α (PGC-1α) protein were observed in human HFpEF muscle, while benefits of exogenous musclin on the oxidative muscle phenotype and exercise capacity were demonstrated in mice [32]. The human observations and experimental treatment effects support different inferences. Additional animal studies investigate irisin-related exercise adaptations and signaling involved in mitochondrial regulation, cellular stress, and muscle atrophy [33,34,35]. These findings identify candidate mechanisms without establishing that the corresponding pathways mediate rehabilitation benefits in humans.

A preclinical HFpEF study reported impaired overload-induced muscle growth that improved with caloric restriction [36]. This model-specific response does not establish that caloric restriction preserves muscle quantity in patients with cardiovascular sarcopenia. In a human HFpEF trial, adding resistance training to caloric restriction and aerobic exercise improved strength normalized to muscle area, while the absolute-strength estimate remained imprecise and skeletal muscle loss was not attenuated [14]. This distinction reinforces the need to evaluate muscle quantity and strength separately when interpreting weight-loss interventions.

**Figure 1:**
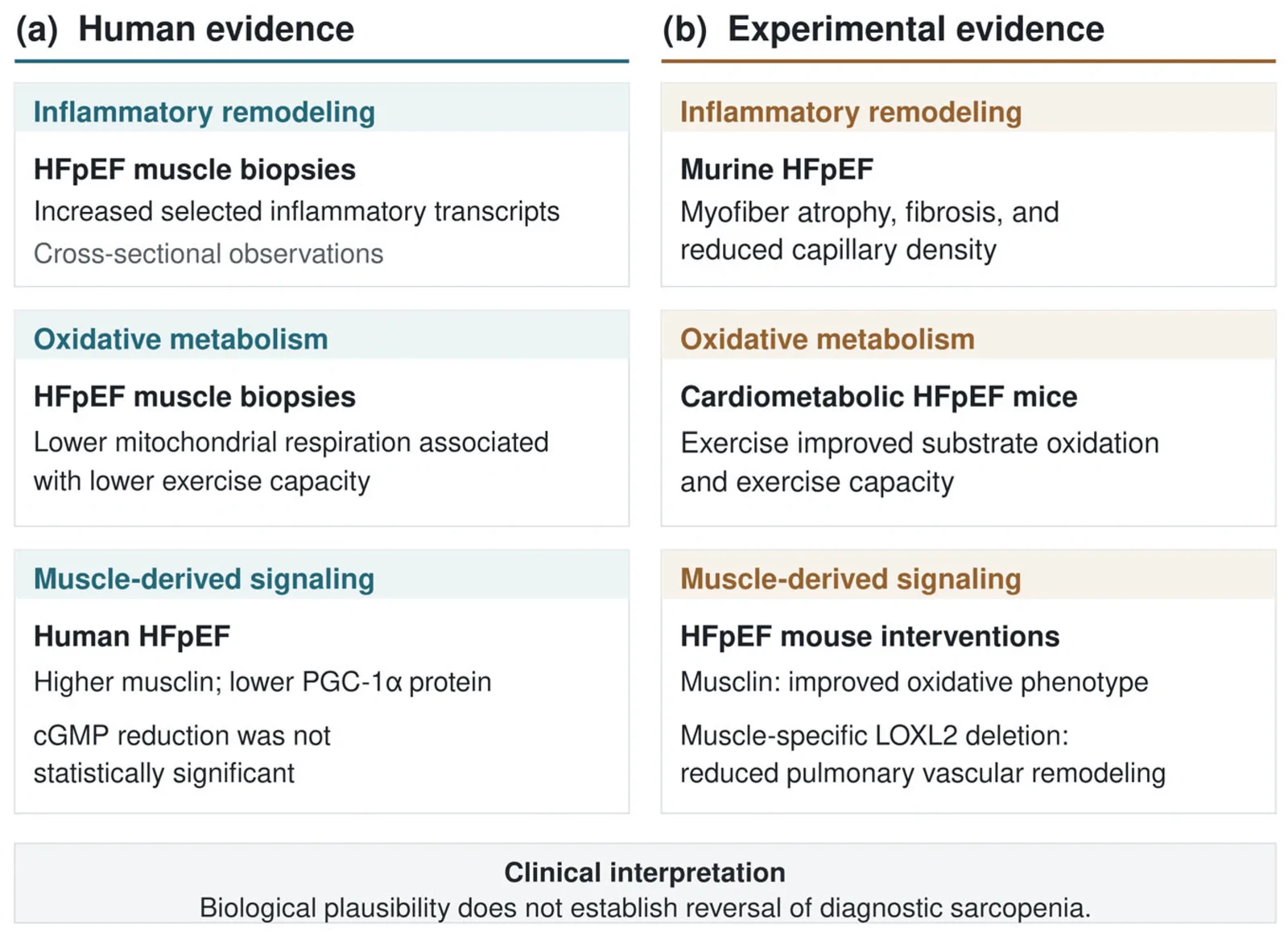
Human and experimental evidence concerning skeletal muscle impairment in cardiovascular disease. (**a**) Human evidence: HFpEF muscle biopsies showed increased expression of selected inflammatory transcripts [10] and lower mitochondrial respiratory capacity associated with exercise intolerance [11]. Increased musclin expression and reduced PGC-1α protein were observed in human HFpEF muscle; the observed decrease in cGMP was not statistically significant [32]. (**b**) Experimental evidence: myofiber atrophy, fibrosis, and reduced capillary density were demonstrated in a murine HFpEF model [10]. Exercise improved skeletal muscle substrate oxidation and exercise capacity in cardiometabolic HFpEF mice [12]. Exogenous musclin improved the oxidative muscle phenotype and exercise capacity in HFpEF mice [32], while muscle-specific Loxl2 deletion reduced pulmonary vascular remodeling in an experimental model of HFpEF-associated pulmonary hypertension [31]. These findings do not establish that modifying the corresponding pathways reverses diagnostically confirmed sarcopenia in humans. HFpEF, heart failure with preserved ejection fraction; PGC-1α, peroxisome proliferator-activated receptor-γ coactivator-1α; cGMP, cyclic guanosine monophosphate; LOXL2, lysyl oxidase-like 2.

Across these pathways, mechanistic findings provide a biological rationale for investigating skeletal muscle dysfunction but do not establish reversal of sarcopenia in humans [31,32,33,34,35,36]. Clinical studies should therefore use an explicit diagnostic framework and report changes in muscle quantity, strength, and functional outcomes separately [1,16]. Figure 1 distinguishes human observations from experimental interventions and highlights the remaining gap between mechanistic plausibility and clinical treatment evidence.

## 3. Clinical Assessment of Sarcopenia in Cardiovascular Disease

### 3.1. Diagnostic Definitions and Muscle Function

Diagnostic definitions influence both participant eligibility and the interpretation of treatment response. EWGSOP2 identifies probable sarcopenia from low strength, confirms the diagnosis through additional evidence of low muscle quantity or quality, and uses poor physical performance to characterize severity [16]. AWGS 2019 requires low muscle mass together with either low strength or low physical performance; impairment in all three domains indicates severe sarcopenia [6]. AWGS 2025 requires concurrent low muscle mass and low strength, provides age-specific thresholds for adults aged 50–64 and ≥65 years, and treats physical performance as an outcome rather than a diagnostic or severity criterion [17]. Table 1 separates these diagnostic requirements from the corresponding measurement thresholds. The GLIS 2024 consensus defines conceptual components but does not supply a numerical operational diagnostic algorithm [1]. In contrast, the REHAB-HF secondary analysis defined functional sarcopenia through strength and performance without confirming low muscle mass; its findings therefore characterize a functional phenotype rather than diagnostic reclassification under a mass-dependent framework [37].

The table presents selected measurements and distinguishes diagnostic requirements from severity and outcome assessments. EWGSOP2 also permits absolute ASM thresholds of <20/<15 kg. Additional EWGSOP2 performance thresholds include TUG ≥ 20 s and inability to complete a 400 m walk or a completion time ≥ 6 min [16]. Five-chair-rise time is a strength assessment under EWGSOP2, whereas AWGS uses it as a physical-performance measure [6,16,17]. AWGS 2025 permits alternative ASM/BMI thresholds: for adults aged ≥65 years, DXA < 0.73/<0.52 and BIA < 0.83/<0.57; for adults aged 50–64 years, DXA < 0.80/<0.55 and BIA < 0.90/<0.63 [17]. ASM/BMI denotes ASM in kg divided by BMI in kg/m^2^. These values must not be interchanged with ASM/height^2^ thresholds. AWGS 2025 does not retain the severe-sarcopenia category [17]. AWGS 2019 applies to older adults aged ≥ 60 or ≥65 years according to country-specific definitions [6]. The age strata shown for AWGS 2025 follow its age-specific measurement table [17]. The GLIS entry refers specifically to the 2024 conceptual consensus and should not be interpreted as a numerical diagnostic algorithm [1].

Physical performance has framework-specific roles. Under EWGSOP2, low performance (gait speed ≤ 0.8 m/s or SPPB ≤ 8, with TUG and 400 m alternatives as above) indicates severe sarcopenia when low strength and low muscle quantity or quality are present [16]. Under AWGS 2019, low performance is defined by 6 m gait speed < 1.0 m/s, SPPB ≤ 9, or five-chair-rise time ≥ 12 s; low muscle mass plus either low strength or low performance establishes sarcopenia, and impairment in all three domains establishes severe sarcopenia [6]. Under AWGS 2025, physical performance is an outcome measure rather than a diagnostic or severity criterion; suggested outcome thresholds are gait speed < 1.0 m/s or five-chair-rise time ≥ 12 s at age ≥ 65 years, and gait speed < 1.2 m/s or five-chair-rise time ≥ 10 s at age 50–64 years [17].

The diagnostic label should identify both the consensus framework and any study-specific adaptation. Replacing the physical-performance component of AWGS 2019 with the six-minute walk test altered prognostic classification in a heart failure cohort, supporting further evaluation of the adaptation rather than its automatic equivalence to the original criteria [38]. The 2026 European Federation of Internal Medicine (EFIM) guideline adaptation similarly emphasizes the practical difficulties of identifying and managing sarcopenia in multimorbid adults and distinguishes evidence-based recommendations from good-practice points where direct evidence is limited [39]. The GLIS outcomes report further separates diagnosis from its consequences, finding stronger evidence for associations with mortality, falls, fractures, and quality of life than for several other functional and healthcare-use outcomes [40].

Analyses from FRAGILE-HF illustrate the prognostic implications of differing operational definitions [41,42,43,44]. Sarcopenia Definitions and Outcomes Consortium (SDOC) criteria and AWGS 2019 criteria showed similar discrimination for mortality, but this finding does not establish diagnostic equivalence [41]. A separate analysis associated a study-specific operationalization of the GLIS concept with prognosis [42]. In 1269 older patients with heart failure, lower handgrip strength was associated with increased two-year mortality, supporting its prognostic value as a directly measured strength parameter [43]. The Ishii score, incorporating age, handgrip strength, and calf circumference, has also been evaluated for sarcopenia case finding and prognostic assessment in this cohort [44]. These publications provide complementary analyses of a shared study population and should not be counted as independent external validation [42,43,44].

Case-finding questionnaires can identify patients requiring direct assessment, but their performance depends on the population and reference definition. In older CVD inpatients, a lower SARC-F threshold improved sensitivity at the cost of limited specificity; this does not establish a universally applicable replacement cutoff [45]. A separate cardiovascular cohort found greater screening discrimination with SARC-CalF than SARC-F, but calf circumference was also used to characterize muscle quantity in the study’s reference assessment [46]. Such methodological dependence should be considered before interpreting a high area under the curve as independent validation of a mass-confirmed diagnosis [46]. Among very old adults hospitalized for acute heart failure, both SARC-F and handgrip strength were associated with short-term mortality, providing prognostic information without replacing comprehensive diagnostic assessment [47].

Handgrip dynamometry requires a standardized measurement protocol, including consistent positioning, equipment, and test procedures, to permit interpretation against the selected diagnostic thresholds [16,17]. Chair-rise testing and gait-speed assessment characterize functional abilities that extend beyond isolated muscle force generation [16,17]. The Short Physical Performance Battery (SPPB) evaluates lower-extremity performance, whereas the six-minute walk test and cardiopulmonary exercise testing (CPET) assess integrated submaximal and maximal exercise capacity, respectively [2,17,48]. These outcomes provide complementary information but should not be substituted for direct assessment of muscle quantity when required by the diagnostic framework [16,17]. Serial evaluation should use consistent methods and distinguish change in a specific performance measure from change in diagnostic sarcopenia status [2,16,17].

Measurement standardization remains incomplete even where consensus thresholds are available. A 2025 Delphi study reached agreement on selected procedures for grip strength, gait speed, and chair-rise testing, but important aspects of protocol and equipment interchangeability remained unresolved [49]. Analyses from the PROFUND-IC registry associated low dynamometric strength and low SPPB scores with adverse prognosis in older patients with acute heart failure [50,51]. These reports arise from a shared registry and address different measurement domains [50,51]. Reduced upper and lower limb strength has also been documented without a corresponding reduction in skeletal muscle mass, reinforcing the need to measure both rather than use one as a proxy for the other [52].

### 3.2. Muscle Quantity and Tissue Characteristics

For diagnostic assessment, the measurement method and normalization index should be specified alongside the selected sarcopenia framework. Dual-energy X-ray absorptiometry (DXA) estimates appendicular lean soft tissue, whereas bioelectrical impedance analysis (BIA) estimates lean or skeletal muscle mass using electrical measurements and prediction equations [16]. These estimates require method-specific interpretation. In patients with heart failure, appendicular lean mass normalized to body mass index and to height squared showed poor agreement in classifying low muscle mass, illustrating that the normalization method can alter classification [53]. A study-derived index should therefore not be substituted for a consensus threshold without appropriate validation.

Fluid status is particularly important when interpreting body composition in heart failure. Extracellular fluid expansion can distort BIA-derived estimates and obscure underlying muscle depletion [53]. An apparently preserved estimate should consequently be interpreted cautiously in patients with clinically significant fluid retention. DXA measurements are also influenced by hydration and should not be regarded as an unaffected alternative [16]. BIA-derived phase angle may provide complementary information, but its interpretation is influenced by fluid distribution and body-cell characteristics [53,54]. Variation in reference definitions and population characteristics further limits the use of a single phase-angle threshold for sarcopenia screening [55].

Regional imaging provides additional information about muscle quantity and tissue characteristics. Computed tomography (CT)-derived cross-sectional area and attenuation describe different aspects of muscle impairment and should not be treated as interchangeable measurements [56,57]. Ultrasound can assess muscle thickness, cross-sectional area, and architecture, although interpretation requires standardized acquisition procedures [58]. In older inpatients, ultrasound-based assessments were associated with functional impairment and frailty, but their discrimination against a BIA-based reference assessment remained limited [59]. These findings support complementary clinical assessment rather than automatic interchangeability among regional imaging, whole-body composition estimates, and operational diagnostic criteria.

For prognostic assessment, imaging findings should be interpreted according to the cardiovascular population and outcome studied. CT-defined muscle depletion has been associated with mortality after transcatheter aortic valve implantation, while higher native skeletal muscle T1 values were associated with heart failure hospitalization or cardiovascular death in HFpEF [60,61]. These observational findings concern risk associations. They do not establish that the imaging measurements are validated markers of rehabilitation response or that modifying them improves clinical outcomes.

For longitudinal monitoring, the distinction between tissue change and measurement variation is essential. Changes in BIA-derived muscle quantity during decongestion should not automatically be attributed to muscle loss or accretion [16,62]. We therefore propose documenting congestion, edema, body weight, and recent decongestion therapy and, whenever feasible, repeating assessments after clinical stabilization using the same device and acquisition protocol. This is a pragmatic approach to interpretation rather than a validated correction for fluid-related error. Serial ultrasound measurements likewise require consistent anatomical landmarks, positioning, and acquisition procedures [58]. Diagnostic reassessment should retain the same operational framework and evaluate its required components, rather than infer recovery from a change in one imaging or body-composition measure [16].

### 3.3. Circulating Biomarkers and Their Clinical Limitations

Circulating biomarkers are being investigated as complementary indicators of muscle impairment, but they are not established substitutes for the assessments required by operational diagnostic criteria [16]. In 458,702 UK Biobank participants, Wilkinson et al. evaluated a creatinine- and cystatin C-based sarcopenia index, reporting areas under the receiver operating characteristic curve of 0.731 (95% CI, 0.710–0.752) in men and 0.711 (95% CI, 0.674–0.749) in women for confirmed sarcopenia [63]. However, confirmed sarcopenia was present in only 0.3% of men and 0.1% of women, and the reference classification combined handgrip strength with BIA-derived muscle quantity [63]. These features warrant caution when transferring the findings to cardiovascular populations. Independent validation across relevant cardiovascular phenotypes and renal-function categories is needed before stand-alone diagnostic use. The index should therefore be considered an adjunct rather than a replacement for the muscle assessments required by the selected diagnostic framework [16,63].

Associations with disease severity or functional status should also be distinguished from prospective prognostic validation. Plasma irisin was associated with systemic inflammation and cardiac and postural dysfunction in chronic heart failure, but these observations did not establish that a change in irisin predicts rehabilitation-induced recovery [64]. Serum myostatin findings were less consistent: one study found no significant difference between patients with heart failure and controls and no significant association with sarcopenia or dynapenia [65]. Such results support marker-specific appraisal rather than treating circulating myokines as a uniform class of validated diagnostic or prognostic tests.

**Figure 2:**
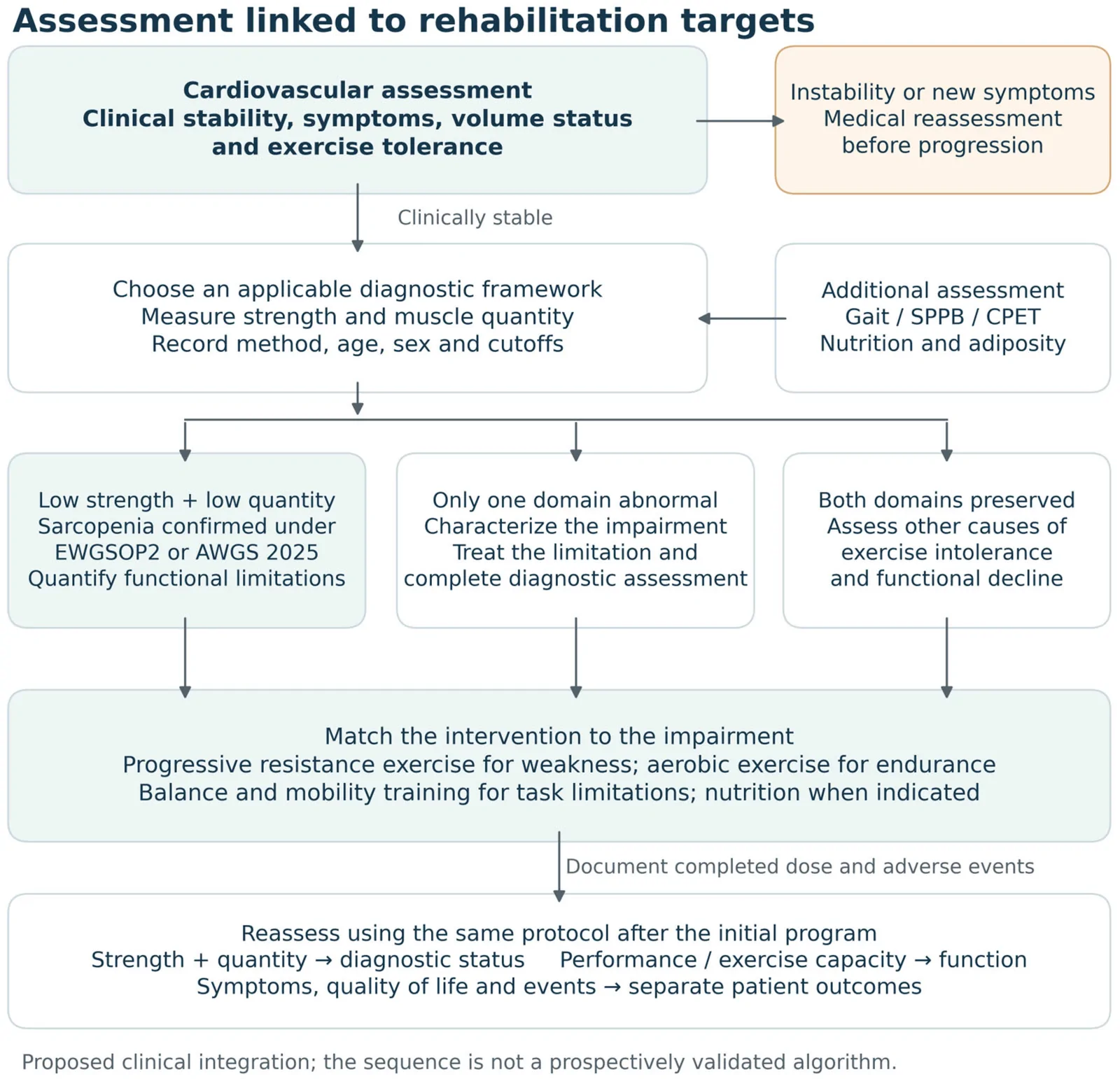
Proposed integration of sarcopenia assessment and exercise rehabilitation in cardiovascular disease. Guideline- and consensus-supported components include framework-specific diagnostic assessment and standardized testing [6,16,17], together with cardiovascular assessment, nutritional evaluation, and individualized exercise planning [2,9,66,67]. The low-strength/low-quantity branch illustrates diagnostic confirmation under EWGSOP2 or AWGS 2025; the diagnostic role of physical performance differs across frameworks and should be interpreted according to Table 1 [6,16,17]. The sequence of assessments, their integration into rehabilitation priorities, and the organization and timing of reassessment represent pragmatic proposals by the authors. Reassessment distinguishes diagnostic status from physical performance, exercise capacity, and patient-relevant outcomes. The complete integrated pathway has not been prospectively validated in patients with CVD-associated sarcopenia. CVD, cardiovascular disease; SPPB, Short Physical Performance Battery; CPET, cardiopulmonary exercise testing. EWGSOP2, revised European Working Group on Sarcopenia in Older People; AWGS, Asian Working Group for Sarcopenia.

For longitudinal application, future studies should determine whether biomarker changes correspond to independently measured changes in muscle quantity, strength, and physical performance and whether they add useful information beyond these assessments. Diagnostic classification, prognostic assessment, and treatment-response monitoring should remain separate purposes. Table 1 summarizes the diagnostic frameworks, while Figure 2 presents the authors’ proposed integration of assessment and rehabilitation.

## 4. Exercise Training Interventions for Sarcopenia in Cardiovascular Disease

Exercise-based rehabilitation addresses exercise intolerance and physical dysfunction in cardiovascular disease, but intervention evidence in patients meeting an explicit sarcopenia definition remains limited [2]. Evidence should therefore be interpreted according to both the cardiovascular population studied and the diagnostic framework used. The coronary artery disease (CAD) pilot trial enrolled participants with low muscle quantity and low handgrip strength [68], whereas the REHAB-HF secondary analysis evaluated functional sarcopenia using the Sarcopenia Definitions and Outcomes Consortium criteria [37]. These reports provide evidence for different diagnostically defined populations; findings under one framework do not establish diagnostic recovery under another. Trials in broader cardiovascular populations without sarcopenia as an enrollment requirement provide indirect evidence for the treatment of diagnostically defined cardiovascular sarcopenia [15,48].

Improvements in muscle strength, muscle quantity, physical performance, and exercise capacity represent distinct outcomes and should be reported separately [14,15,48]. In this review, diagnostic recovery refers to no longer meeting the same operational definition used at baseline, following reassessment of its required components. Improvement in a performance test alone does not establish recovery under a definition that also requires assessment of muscle quantity [16]. Intervention findings are interpreted according to the enrolled population and diagnostic framework, the outcome domain assessed, and the duration of observation. Table 2 summarizes the principal intervention outcomes, while Table S2 presents selected trial populations, diagnostic eligibility, and assessment periods. Where follow-up is available, effects observed during an active intervention are distinguished from persistence after its completion.

### 4.1. Aerobic Exercise and Muscle Metabolic Adaptation

OptimEx-Clin randomized 180 patients with stable HFpEF without requiring a sarcopenia diagnosis [48]. At three months, peak oxygen uptake favored high-intensity interval training and moderate continuous training over physical activity advice by 1.5 mL/kg/min (95% CI, 0.4–2.7) and 2.0 mL/kg/min (95% CI, 0.9–3.1), respectively [48]. Both point estimates were below the prespecified clinically important difference of 2.5 mL/kg/min, although the confidence intervals included larger effects. Interval training was not superior to continuous training, and between-group comparisons of peak oxygen uptake were not statistically significant at 12 months [48]. These findings concern exercise capacity and do not establish diagnostic recovery from sarcopenia.

Evidence from HFpEF trials supports distinguishing exercise capacity from cardiac and patient-reported outcomes. In an OptimEx echocardiographic substudy, changes in exercise diastolic function did not explain the improvement in peak oxygen uptake [74]. Syntheses report improvements in exercise capacity and selected quality-of-life measures, with findings differing across intervention comparisons and outcome instruments [75,76]. Network rankings from broader heart failure populations and strength findings from older-adult high-intensity interval training studies provide indirect guidance; they do not establish that a particular modality is superior for patients with diagnostically confirmed cardiovascular sarcopenia [77,78].

### 4.2. Resistance Exercise and Muscle Function

Resistance exercise targets muscular strength through progressive loading and is recommended for appropriately selected individuals with CVD [66]. The American Heart Association (AHA) scientific statement describes benefits for strength, body composition, and cardiovascular health, while emphasizing individualized prescription [66]. The EAPC statement likewise identifies resistance exercise as an important component of management for cardiovascular patients with sarcopenia [2]. Initial loading and progression should be adapted to baseline functional capacity and cardiovascular status within a supervised or otherwise appropriately monitored program [2]. Repeated assessment of strength and muscle quantity is necessary because functional adaptation and muscle tissue preservation are distinct outcomes [14].

In the 20-week trial of 88 patients with obesity-related HFpEF, the adjusted incremental effect of resistance training on knee-extensor strength was 6.4 Nm (95% CI, −0.1 to 12.9; *p* = 0.053) [14]. Strength normalized to muscle area favored resistance training by 0.07 Nm/cm^2^ (95% CI, 0.00–0.13; *p* = 0.043), whereas the between-group difference in skeletal muscle mass was −0.1 kg (95% CI, −1.0 to 0.8) [14]. Both groups received caloric restriction and aerobic training, and 77 participants completed the trial. The strength estimate remained imprecise, and these findings do not establish muscle preservation or durability beyond 20 weeks. Sarcopenia was not an enrollment requirement [14].

The assessor-blinded pilot trial enrolled 40 older adults with coronary artery disease and sarcopenia defined by low BIA-derived muscle quantity and low handgrip strength [68]. Reported immediate standardized effects favored exercise for SPPB performance (Hedges’ g = 0.637) and handgrip strength (g = 0.552). The sample-size rationale cited an SPPB change exceeding one point as clinically important, but standardized effects alone do not establish attainment of that threshold. Inconsistencies between published SPPB coefficients and confidence intervals further limit precise interpretation. Between-group functional benefits were not demonstrated three months after intervention completion, and no statistically significant muscle-mass benefit was reported. These findings provide preliminary evidence of short-term functional benefit rather than sustained diagnostic recovery [68].

Low-load approaches may improve accessibility, but the load, effort, supervision, and patient selection remain relevant. A review discusses low-intensity resistance exercise as an option within cardiac rehabilitation, drawing on mechanistic and clinical evidence of varying directness [79]. A small cardiac-surgery pilot combined low-load exercise with blood-flow restriction and reported improvements in muscle measures without observed adverse effects under medical monitoring [80]. Its small sample does not establish safety across all cardiovascular risk groups or justify unsupervised application [80].

Evidence from older adults with sarcopenia supports resistance-based treatment but should be identified as indirect when applied to CVD. Network and conventional meta-analyses generally support improvements in strength and physical performance, with less consistent changes in muscle quantity [81,82]. Broader heart failure rehabilitation evidence addresses distinct clinical and quality-of-life outcomes [83]. A 2025 resistance-training meta-analysis similarly found endpoint-dependent benefits, no significant pooled gain in appendicular muscle mass, and uncertainty about whether average improvements exceeded clinically important thresholds [84]. Consequently, statistical significance and clinical importance should both inform interpretation of an exercise response [84].

### 4.3. Multicomponent Rehabilitation

REHAB-HF randomized 349 older adults hospitalized with acute decompensated heart failure to a tailored intervention targeting strength, balance, mobility, and endurance or an attention-control group receiving usual care [15]. At three months, the adjusted between-group difference in SPPB score was 1.5 points (95% CI, 0.9–2.0), whereas six-month rehospitalization and mortality were not significantly reduced [15]. The principal demonstrated benefit was improved physical performance; this finding does not establish recovery from sarcopenia defined by muscle quantity and strength.

A secondary analysis classified participants using the Sarcopenia Definitions and Outcomes Consortium functional criteria, combining low handgrip strength and slow gait speed without requiring low muscle quantity [37]. These findings inform rehabilitation in SDOC-defined functional sarcopenia, but cannot be directly translated into recovery under definitions requiring low muscle quantity. The subgroup analysis derives from the same randomized trial and should not be treated as independent replication [15,37].

Acute-phase rehabilitation findings differ according to the population, intervention, and assessment period. ACTIVE-ADHF enrolled 91 hospitalized patients without physical frailty and reported an additional 30.9 m improvement in six-minute walk distance at the short-term, approximately two-week assessment (95% CI, 4.8–57.0) [69]. The interval includes both small and larger walking-distance benefits, and this finding does not establish persistence after program completion. In contrast, Wu et al. studied 120 critically ill patients receiving rehabilitation during their cardiac intensive-care unit (CICU) stay [70]. The discharge SPPB median between-group difference was 1.0 point (95% CI, 0.0–2.0), and the six-month rehospitalization hazard ratio was 1.00 (95% CI, 0.51–1.99); neither coprimary outcome demonstrated a statistically significant benefit [70]. The rehospitalization interval remains compatible with clinically important benefit or harm and does not establish equivalence. These trials should therefore be interpreted within their respective clinical settings rather than used to infer a common rehabilitation effect [69,70].

SPRINTT provides longer-term evidence in an older population selected for physical frailty and trial-defined sarcopenia rather than concomitant cardiovascular disease [85]. Among 1519 randomized participants, the prespecified primary comparison included 1205 participants with baseline SPPB scores of 3–7. Over a mean follow-up of 26.4 months, mobility disability occurred in 46.8% of the multicomponent intervention group and 52.7% of controls (hazard ratio, 0.78; 95% CI, 0.67–0.92) [85]. These findings support mobility-disability prevention during a continued physical activity and nutritional counseling program. Persistence after program completion and treatment effects specifically in cardiovascular sarcopenia remain separate questions.

Other community and nursing-home studies provide contextual evidence concerning functional exercise programs, but their populations do not directly estimate treatment effects in cardiovascular sarcopenia [86,87,88]. Additional reports address combined training in HFrEF, rehabilitation after transcatheter aortic valve replacement (TAVR), prehabilitation before cardiac surgery, and functional electrical stimulation in chronic heart failure [89,90,91,92]. These studies broaden the evidence concerning rehabilitation delivery across clinical settings; their differing populations and outcome domains preclude interpreting them collectively as evidence of diagnostic recovery from sarcopenia.

### 4.4. Nutritional Support and Weight Management

Nutritional management should distinguish prevention or treatment of malnutrition, improvement in functional outcomes, and preservation of muscle quantity as separate therapeutic goals. The European Society for Clinical Nutrition and Metabolism (ESPEN) geriatric guideline recommends routine malnutrition screening and individualized nutritional counselling, food modification, and oral supplementation when indicated [93]. In the prespecified EFFORT heart failure analysis, 645 hospitalized patients at nutritional risk received individualized nutritional support or standard hospital food [71]. Thirty-day mortality was 8.4% versus 14.8%, respectively (adjusted odds ratio, 0.44; 95% CI, 0.26–0.75) [71]. The intervention addressed energy, protein, and micronutrient goals together, so its effects cannot be attributed to protein supplementation alone or to an exercise–nutrition interaction. Moreover, eligibility was based on nutritional risk rather than a required sarcopenia diagnosis. These findings support individualized nutritional care but do not establish diagnostic recovery from sarcopenia [71].

Nutritional risk, cachexia, and sarcopenia should be recorded separately. A heart failure cohort using Asian Working Group for Cachexia (AWGC) 2023 criteria demonstrated substantial overlap with frailty and sarcopenia, and a cohort meta-analysis associated cachexia with adverse prognosis [94,95]. A review describes how congestion, reduced intake, and chronic disease complicate nutritional assessment [96]. An outpatient study obtained substantially different estimates depending on the nutritional screening or diagnostic tool, while another heart failure cohort found only a weak correlation between nutritional risk and SPPB [97,98]. No single nutrition score should therefore be assumed to represent muscle strength or physical performance [97,98].

Smaller nutritional studies provide preliminary supportive evidence with important design limitations. An open-label randomized pilot of high-energy, high-protein supplementation reported improvements in nutritional and functional measures during hospitalization, but was not powered to establish durable clinical-event benefit [99]. An observational study of essential amino acid supplementation in older patients with HFrEF and sarcopenia reported favorable changes over follow-up, but concurrent optimized medical treatment and the nonrandomized design limit attribution to supplementation [100]. A review of nutritional interventions in older frail patients with heart failure identified a small, heterogeneous evidence base, supporting individualized care rather than a universal supplement regimen [101].

Evidence for protein supplementation during resistance exercise comes largely from older populations not specifically selected for cardiovascular disease [102,103]. Although pooled findings suggest benefits for selected muscle outcomes, effects vary by comparator, supplement, and outcome. The whey-protein review reported small effects and rated the certainty of evidence as low to very low; this assessment was performed by the review authors rather than within the present critical review [103]. A separate β-hydroxy-β-methylbutyrate (HMB) meta-analysis found improved gait speed without statistically significant benefits for muscle-mass index, grip strength, or fat-free mass [104]. Functional improvement should therefore not be interpreted as uniform muscle preservation. These findings provide indirect guidance but do not establish a universal supplement regimen or a synergistic exercise–nutrition effect in cardiovascular sarcopenia [103,104].

Intentional weight reduction addresses a different clinical problem from nutritional repletion. ESPEN recommends considering weight-reducing diets in obese older adults with weight-related health problems after individualized assessment of benefits and risks, with exercise whenever feasible [93]. In STEP-HFpEF, semaglutide improved symptoms and physical limitations and increased six-minute walk distance at 52 weeks compared with placebo (between-group difference, 20.3 m; 95% CI, 8.6–32.1) [105]. In SUMMIT, tirzepatide reduced the composite risk of cardiovascular death or worsening heart failure over a median follow-up of 104 weeks (hazard ratio, 0.62; 95% CI, 0.41–0.95) [106]. These trials establish benefits in obesity-related HFpEF but do not demonstrate preservation of muscle quantity or reversal of diagnostically confirmed sarcopenia.

Muscle preservation during weight reduction requires separate evaluation. In the trial adding resistance training to caloric restriction and aerobic exercise, skeletal muscle mass declined in both groups, without demonstrated attenuation from added resistance training [14]. Because protein provision was not independently randomized, this comparison does not establish the optimal protein strategy or whether a different protein intervention would modify the response. Adequate protein provision and resistance exercise should therefore be considered within an individualized plan rather than assumed to prevent muscle depletion during every weight-loss intervention [2,93].

Clinical planning should specify whether the nutritional goal is repletion, functional improvement, or muscle preservation. Energy and protein provision should be individualized according to nutritional status, renal function, clinical condition, and treatment tolerance, while fluid and sodium management should be coordinated with heart failure care [71,93]. Protein provision should also be interpreted alongside total energy intake and the exercise actually completed [2,93]. Reassessment should distinguish changes in dietary intake and body weight from changes in muscle quantity, strength, and physical performance, with attention to concurrent fluid changes [93].

### 4.5. Adherence, Frailty, and Rehabilitation Delivery

**Figure 3:**
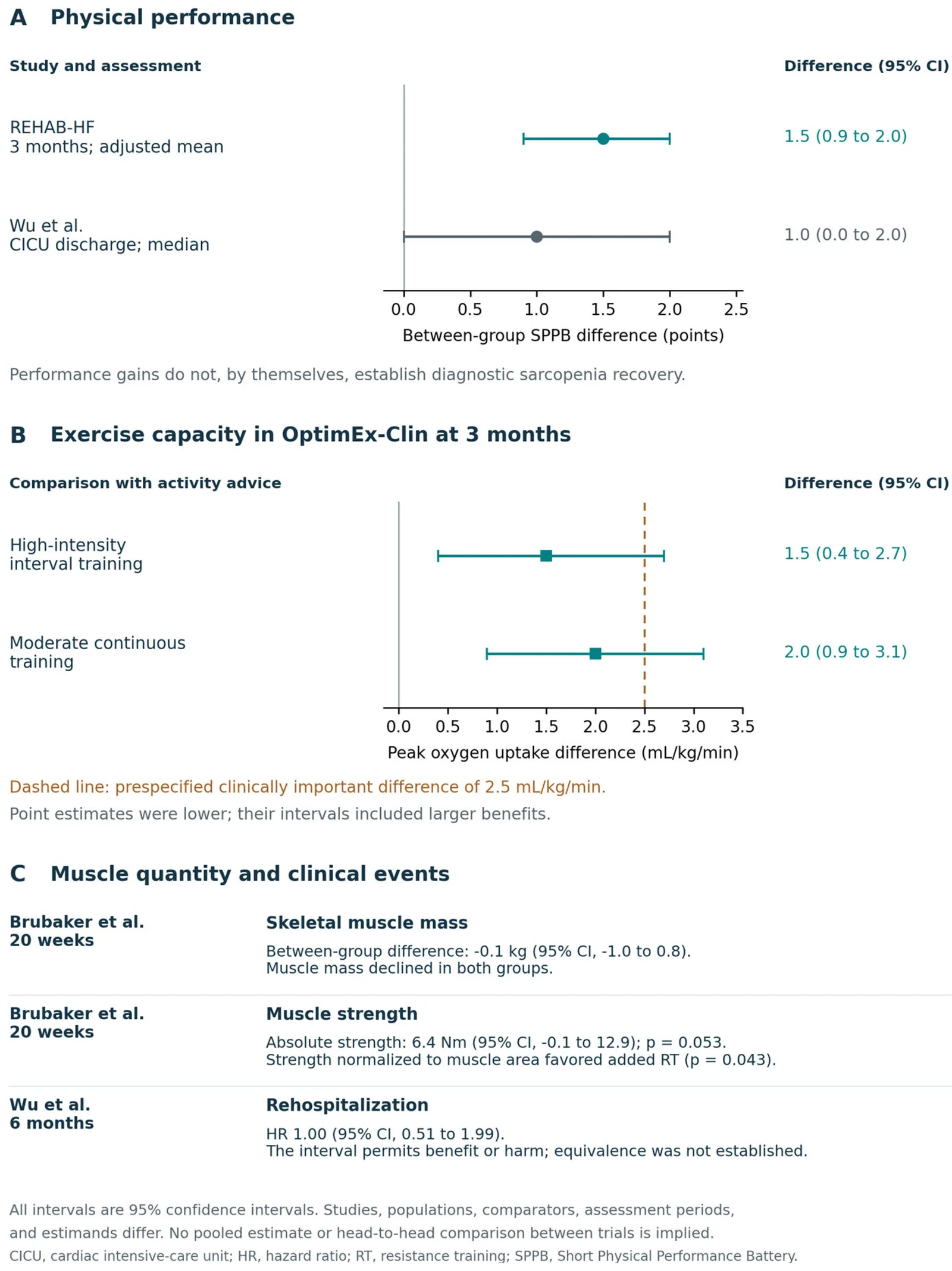
Outcome-specific interpretation of selected randomized trials. (**A**) SPPB effects in REHAB-HF at three months and in the Wu et al. CICU trial at discharge; the former is an adjusted mean difference and the latter a median difference [15,70]. (**B**) Peak oxygen uptake differences versus physical activity advice in OptimEx-Clin at three months [48]. The dashed line denotes the prespecified clinically important difference of 2.5 mL/kg/min; both point estimates were lower, while their intervals included larger effects. (**C**) Muscle quantity and clinical events require separate assessment: resistance training during weight reduction in HFpEF favored strength normalized to muscle area, while the absolute-strength estimate remained imprecise and muscle mass declined in both groups [14], and the imprecise rehospitalization estimate in the CICU trial did not establish equivalence [70]. All plotted intervals are 95% CIs. These trials differ in population, comparator, follow-up, and estimand; no pooled or head-to-head cross-trial comparison is implied. CI, confidence interval; SPPB, Short Physical Performance Battery; CICU, cardiac intensive-care unit; HFpEF, heart failure with preserved ejection fraction.

Adherence determines how much of the prescribed exercise is completed. Within the REHAB-HF intervention group, greater attendance was associated with larger improvements in physical function and health-related quality of life [72]. Attendance was not randomized and also reflects health status and capacity to participate, so this association cannot establish a causal exercise dose–response relationship [72]. Recording completed sessions, interruptions, symptoms, and changes in concomitant treatment helps interpret individual response and adapt delivery [9,72]. Table 2 compares selected trials, including direct evidence in confirmed sarcopenia, and Figure 3 displays key effect estimates and their uncertainty.

Frailty influences both access to rehabilitation and the interpretation of response. Meta-analyses and reviews, including a scoping review, identify substantial variation in frailty tools, program content, and barriers to completion [107,108,109]. A retrospective acute heart failure cohort associated frailty with a lower probability of inpatient SPPB improvement, whereas REHAB-HF found a larger randomized treatment response in frail than prefrail participants [73,110]. These findings address different designs and treatment settings and should not be reduced to a claim that frailty invariably predicts either response or nonresponse [73,110].

Access and participation also differ by frailty. In Medicare claims, greater frailty was associated with lower cardiac rehabilitation use, while participation was associated with lower mortality across frailty strata [111]. Residual differences in access, clinical stability, and capacity to attend remain plausible explanations for part of this nonrandomized association [111]. Practical adaptation should address transport, cognitive and emotional barriers, assistance requirements, and tolerable exercise progression, as discussed in contemporary reviews of frailty-focused cardiovascular rehabilitation [108,112].

Home and remote delivery can broaden access, but efficacy depends on the intervention rather than the communication technology alone. Meta-analyses generally support gains in exercise capacity compared with no rehabilitation, with inconsistent quality-of-life findings and no clear advantage over center-based care [113,114]. In a randomized trial among patients declining standard outpatient rehabilitation, an initial videoconference exercise program did not produce significant between-group improvements in physical activity or peak oxygen uptake at two years [115]. This neutral result highlights the distinction between offering remote sessions and achieving sustained behavioral change [115].

Implementation studies provide additional information about feasibility and reach. SCOT:REACH-HF reported improved health-related quality of life after rollout of a facilitated home program, but its implementation design differs from a randomized efficacy comparison [116]. A pilot of an interactive indoor cycling device assessed short-term physiological responses in a small, selected male CVD sample; it did not establish long-term adherence, sarcopenia recovery, or clinical-event reduction [117]. Digital tools should consequently be evaluated for accessibility, completed exercise dose, retention, and sustained outcomes in the intended clinical population [116,117].

## 5. Clinical Implications and Future Directions

### 5.1. Clinical Prescription and Reassessment

The assessment and rehabilitation pathway in Figure 2 is an author-proposed synthesis of diagnostic consensus recommendations and cardiac rehabilitation principles [2,9,16]. Consensus-supported components include assessment using an explicit diagnostic framework, individualized exercise prescription, and nutritional evaluation [2,9,16]. Their arrangement into the proposed sequence and the suggested reassessment schedule reflect our pragmatic integration of the available evidence. The complete pathway has not been prospectively validated in patients with CVD-associated sarcopenia, and its clinical effectiveness as an integrated care strategy remains to be tested. Its application therefore requires adaptation to cardiovascular stability, volume status, comorbidities, baseline function, and the patient’s ability to participate [2,9].

Trial protocols illustrate feasible exercise doses in defined populations. In stable older adults with obesity-related HFpEF, Brubaker et al. added approximately 20 min of resistance exercise to aerobic training three days weekly for 20 weeks [14]. Resistance work began at 20–30% of one-repetition maximum, using one set of 8–12 repetitions, and progressed after four weeks to 40–50% with two sets; loads increased by approximately 5% when participants exceeded 12 repetitions in the second set during two consecutive sessions [14]. One-repetition maximum was reassessed every four weeks. This is a trial-tested progression for a selected stable population, not a universal starting prescription for acute heart failure or severe frailty.

For patients recovering from acute decompensation, REHAB-HF used individualized strength, balance, mobility, and endurance tasks across a target of 36 outpatient sessions over 12 weeks [15]. Progression depended on performance in each domain rather than on an identical load for all participants. In confirmed sarcopenia with stable CAD, the pilot resistance program used two 60 min sessions weekly for 12 weeks [68]. These schedules provide practical examples, but differences in stability, supervision, and eligibility must be preserved when adapting them. The primary goal is completion of an appropriate, progressive dose, documented alongside symptoms and interruptions [15,66,72].

Aerobic dose should likewise reflect capacity and cardiovascular status. OptimEx-Clin prescribed three 38 min interval sessions or five 40 min continuous sessions weekly, with three months of supervised training followed by home training [48]. The trial did not establish superiority of interval training, supporting selection according to tolerance, preference, and the likelihood of continued participation [48]. Heart rate, blood pressure, symptoms, and, where indicated, rhythm monitoring support supervised progression; new chest discomfort, presyncope, worsening congestion, or disproportionate dyspnea requires clinical reassessment rather than automatic escalation [66,67].

Nutritional assessment and appropriate individualized support should accompany exercise planning [2,9]. Reassessment should retain the original diagnostic framework and measurement protocols, with attention to clinically comparable hydration status when interpreting muscle quantity [16]. An assessment at approximately 12 weeks is offered as a practical example informed by selected rehabilitation trials [15,68]; it is not an established optimal interval for CVD-associated sarcopenia. Timing should be individualized according to the clinical course and intervention, with earlier review when clinical status changes [9]. Follow-up after supervised training is also needed because short-term improvement does not establish persistence of benefit or sustained diagnostic recovery [48,68].

### 5.2. Priorities for Clinical and Mechanistic Trials

The most informative future trials will recruit patients using an explicit sarcopenia definition, document the cardiovascular phenotype and baseline muscle components, and retain the same assessment methods during follow-up. Existing studies differ in eligibility, clinical setting, intervention duration, and outcome selection, limiting transfer of an average treatment effect between populations [14,73]. Trial reports should distinguish prescribed from completed exercise, describe progression and adverse events, and account for cointerventions. Factorial or otherwise suitable designs could separate nutritional and exercise contributions. Follow-up after supervised treatment remains important because the CAD pilot did not demonstrate sustained between-group functional benefits three months after program completion [68], and the molecular changes in the OptimEx biopsy substudy were no longer evident at 12 months [13].

Human muscle findings concerning inflammatory expression [10] and mitochondrial respiration [11], together with experimental evidence concerning muscle–pulmonary vascular signaling [31], identify candidate pathways for further clinical investigation. Repeated tissue or circulating measurements can test whether biological changes precede or accompany changes in muscle strength and function; formal mediation analyses would require designs and assumptions beyond a simple pre–post association. Imaging and biomarker studies should establish reproducibility, responsiveness, and incremental clinical utility in the intended cardiovascular population before these measurements are used to guide treatment [56,57].

The principal evidence gap concerns diagnostic recovery and its clinical consequences. In obesity-related HFpEF, adding resistance exercise to caloric restriction and aerobic exercise did not demonstrate preservation of skeletal muscle mass [14], whereas REHAB-HF improved physical performance without demonstrating a reduction in six-month clinical events [15]. Conversely, broader pooled heart failure evidence supports reduced hospital admission and improved health-related quality of life with exercise-based cardiac rehabilitation [83]. These findings are compatible because the populations and outcomes differ. Trials in confirmed sarcopenia should therefore assess the defining muscle components, sustained functional independence, and clinical events as separate outcomes, rather than assume that change in one domain validates the others [16].

### 5.3. Limitations of the Review

This review used targeted retrieval and relevance assessment rather than exhaustive systematic screening. The revision-stage PubMed update was limited to English-language records and focused searches in a single database, introducing potential retrieval, selection, and language bias. No formal systematic risk-of-bias or certainty assessment was undertaken, limiting comparative ranking of interventions.

Diagnostic thresholds, cardiovascular phenotypes, measurement methods, treatment doses, and follow-up periods differ across the included reports, and multiple publications from shared cohorts increase the risk of overstating independent replication. Many exercise trials enrolled patients with frailty or exercise intolerance without requiring an explicit sarcopenia diagnosis, limiting direct inference about treatment of diagnostically defined cardiovascular sarcopenia. Findings based on functional definitions should be interpreted within those definitions. Studies in other older populations provide indirect clinical guidance, whereas experimental studies provide mechanistic support. Accordingly, the proposed assessment pathway and prescription examples synthesize available evidence but require adaptation and prospective validation in explicitly defined cardiovascular sarcopenia populations.

## 6. Conclusions

Exercise rehabilitation can improve selected muscle and functional outcomes in cardiovascular populations, but direct evidence for sustained diagnostic recovery in patients with confirmed CVD-associated sarcopenia remains limited [2,68]. Muscle strength, muscle quantity, physical performance, and exercise capacity should be interpreted as distinct outcomes. Strength normalized to muscle area can improve without preservation of muscle quantity, and improvements in physical performance do not by themselves establish a reduction in hospitalization or mortality [14,15].

Clinical assessment should use an explicit diagnostic framework and guide individualized exercise and nutritional management [2,9,16]. The pathway proposed in this review integrates these principles, but requires prospective evaluation as a complete care strategy. Future trials should enroll patients with a prespecified sarcopenia diagnosis, reassess the required diagnostic components, and extend follow-up beyond supervised training to determine whether benefits persist [2,16,68]. Muscle outcomes, functional independence, hospitalization, and mortality should be evaluated separately, with sufficient follow-up and statistical power to establish their clinical significance.

## Figures and Tables

**Table 1:** Selected diagnostic requirements and measurement thresholds relevant to cardiovascular rehabilitation.

Framework and Population	Diagnostic Requirement	Low Muscle Strength	Low Muscle Quantity: ASM/Height^2^, kg/m^2^
EWGSOP2 Older adults [16]	Low strength: probable sarcopenia. Additional low muscle quantity or quality: confirmed sarcopenia.	Handgrip < 27/<16 kg; alternatively, five chair rises >15 s.	<7.0/<5.5. Absolute ASM alternatives are provided below.
AWGS 2019 Older Asian adults [6]	Low muscle mass AND either low strength OR low physical performance.	Handgrip < 28/<18 kg.	DXA: <7.0/<5.4; BIA: <7.0/<5.7.
AWGS 2025 Age ≥ 65 years [17]	Low muscle mass AND low strength.	Handgrip < 28/<18 kg.	DXA: <7.0/<5.4; BIA: <7.0/<5.7.
AWGS 2025 Age 50–64 years [17]	Low muscle mass AND low strength.	Handgrip < 34/<20 kg.	DXA: <7.2/<5.5; BIA: <7.6/<5.7.
GLIS conceptual definition [1]	Conceptual components include muscle mass, muscle strength, and muscle-specific strength; no operational cutoff algorithm is specified.	Numerical thresholds not specified.	Numerical thresholds not specified.

Values are presented as men/women. ASM, appendicular skeletal muscle mass; BIA, bioelectrical impedance analysis; EWGSOP2, revised European Working Group on Sarcopenia in Older People; AWGS, Asian Working Group for Sarcopenia; GLIS, Global Leadership Initiative in Sarcopenia.

**Table 2:** Selected intervention trials and the interpretation of muscle, functional, and clinical outcomes.

Trial and Eligibility	Intervention and Comparator	Principal Findings	Clinical Interpretation and Feasibility
CAD pilot [68] *n* = 40; age ≥ 60 Low mass + low grip strength	12-week resistance-based exercise, two 60 min sessions weekly, versus usual care.	Strength and SPPB improved immediately after treatment; no significant muscle-mass benefit. Between-group functional benefits were not demonstrated three months after program completion.	Direct evidence in confirmed sarcopenia; small pilot. Attendance 94%; no serious adverse events reported. Durability remains unresolved.
Brubaker et al. [14] *n* = 88 Stable obesity-related HFpEF	20 weeks: CR + AT with or without RT; supervised exercise three days weekly.	Knee-extensor strength difference: 6.4 Nm (95% CI, −0.1 to 12.9; *p* = 0.053). Strength per muscle area: 0.07 Nm/cm2 (95% CI, 0.00–0.13; *p* = 0.043). Muscle mass declined in both groups.	Indirect evidence for confirmed sarcopenia. Seventy-seven of 88 participants completed the trial; no study-related serious adverse events were reported. Muscle preservation was not demonstrated.
REHAB-HF [15] *n* = 349 Older adults hospitalized with acute HF	Individualized strength, balance, mobility, endurance; target 60 min, three times weekly, 12 weeks after discharge; usual-care control.	Three-month SPPB difference 1.5 points (95% CI 0.9–2.0). No significant handgrip benefit or reduction in six-month rehospitalization or mortality.	Physical-performance benefit; muscle quantity was not assessed, so recovery under definitions requiring low muscle quantity could not be established.
OptimEx-Clin [48] *n* = 180 Stable HFpEF	Interval: 3 × 38 min/week; continuous: 5 × 40 min/week; activity-advice control. Three supervised months, then nine home months.	Three-month peak oxygen uptake: interval +1.5 (0.4–2.7), continuous +2.0 (0.9–3.1) mL/kg/min versus advice. No significant 12-month differences.	Point estimates below the 2.5 mL/kg/min threshold; intervals include larger effects. Sarcopenia not required. Three-month assessment: 166/180 participants.
ACTIVE-ADHF [69] *n* = 91 Acute HF without physical frailty	Early intensive exercise during hospitalization versus usual care.	Additional change in six-minute walking distance: 30.9 m (95% CI 4.8–57.0).	Applies to a selected nonfrail acute-HF population. The result is a walking-capacity outcome, not evidence of diagnostic sarcopenia recovery.
Wu et al. [70] *n* = 120 Critically ill acute HF after stabilization	Early rehabilitation during the CICU stay versus usual care.	Discharge SPPB median difference 1.0 (0.0–2.0), *p* = 0.16. Six-month rehospitalization HR 1.00 (0.51–1.99).	No significant coprimary benefit; imprecision does not establish equivalence. The short CICU intervention differs from sustained post-discharge rehabilitation.
EFFORT HF analysis [71] *n* = 645 Hospitalized HF with nutritional risk	Individualized nutritional support versus standard hospital food.	Thirty-day mortality: 8.4% versus 14.8%; adjusted odds ratio, 0.44 (95% CI, 0.26–0.75)	Supports treatment of nutritional risk. Does not isolate protein effects or test interaction with exercise; sarcopenia was not required.

CI, confidence interval; CAD, coronary artery disease; HF, heart failure; HFpEF, heart failure with preserved ejection fraction; SPPB, Short Physical Performance Battery; RT, resistance training; AT, aerobic training; CR, caloric restriction; CICU, cardiac intensive-care unit; HR, hazard ratio. Values describe the reported comparison and follow-up; blank effect estimates have not been reconstructed from incomplete reports. Attendance and safety measures are not standardized across trials. The CAD trial used low BIA-derived ASM/height^2^ (<7.0/<5.7 kg/m^2^ in men/women) plus low grip strength (<28/<18 kg), consistent with its registered eligibility criteria (NCT05497687) [68]. REHAB-HF analyses of functional sarcopenia, frailty, and attendance derive from the same trial [37,72,73], and the OptimEx biopsy report is a nested substudy of the parent trial [13,48]. EFFORT is a nutritional comparison included to contextualize nutritional risk, not an exercise trial.

## Data Availability

No new data were created or analyzed in this review. Data sharing is not applicable to this article.

## Supplementary Materials

The following supporting information can be downloaded at: https://www.mdpi.com/article/10.3390/jcm15218637/s1, Table S1: Targeted literature retrieval during revision and principles guiding evidence selection; Table S2: Population eligibility, diagnostic basis, and applicability of selected rehabilitation studies to cardiovascular sarcopenia.
