# Supplementary material for: Clinical Assessment and Exercise Rehabilitation of Sarcopenia in Cardiovascular Disease
Source: J Clin Med. 2026 Nov 5;15(21):8637. doi: 10.3390/jcm15218637 (PMC13763716; doi:10.3390/jcm15218637)
Supplement: Supplementary file 1 [file jcm-15-08637-s001.zip › jcm-4641540-supplementary.pdf]

Supplementary Table S1

Table S1. Targeted literature retrieval during revision and principles guiding evidence selection.

A. Search conditions

Parameter	Recorded information
Database	PubMed
Search date	27 October 2026 (Korea Standard Time)
Publication period	1 January 2021 to 27 October 2026; Date - Publication field
Language	English
Search fields and filters	Title and Title/Abstract fields as specified in Part C; no species, age, or study-design filters
Retrieval and deduplication	All returned identifiers retrieved; duplicate records identified by PMID
Search results	321 query entries; 292 unique PubMed records
Scope of the update	Revision-stage update supplementing the existing bibliography; earlier foundational consensus documents and pivotal trials retained where relevant

B. Evidence selection principles

Item	Principle
Relevance assessment	Titles inspected for relevance; selected available abstracts examined to clarify study design, population, and outcomes
Prioritization	Original consensus documents and clinical investigations relevant to the diagnostic, mechanistic, or rehabilitation questions
Clinical applicability	Studies meeting an explicit sarcopenia definition distinguished from broader cardiovascular or functional populations; other older populations considered indirect evidence
Mechanistic interpretation	Human observations and experimental interventions considered separately
Related publications	Reports from shared trials or cohorts treated as related evidence rather than independent replication
Reports not prioritized or used for efficacy	Reports without relevant diagnostic, mechanistic, or rehabilitation findings not prioritized; protocols without intervention results not used to establish treatment efficacy
Methodological limits	Targeted, non-exhaustive retrieval and relevance assessment; no formal systematic risk-of-bias assessment, certainty grading, or meta-analysis

C. Complete PubMed search strategies

The complete executed queries below include the language and publication-date limits. Results were sorted by publication date, and all returned identifiers were retrieved.

ID	Topic and complete search query	Records
Q1	**Diagnostic frameworks**<br>(sarcopenia[Title] AND (consensus[Title] OR definition*[Title]) AND (EWGSOP2[Title/Abstract] OR "Asian Working Group"[Title/Abstract] OR "Global Leadership Initiative"[Title/Abstract] OR "Sarcopenia Definitions"[Title/Abstract])) AND english[Language] AND ("2021/01/01"[Date - Publication] : "2026/10/27"[Date - Publication])	69
Q2	**Clinical assessment**<br>((sarcopenia[Title] OR "skeletal muscle"[Title]) AND ("heart failure"[Title] OR "cardiovascular disease"[Title]) AND (diagnos*[Title/Abstract] OR assess*[Title/Abstract] OR bioimpedance[Title/Abstract] OR "bioelectrical impedance"[Title/Abstract] OR "muscle quantity"[Title/Abstract])) AND english[Language] AND ("2021/01/01"[Date - Publication] : "2026/10/27"[Date - Publication])	162
Q3	**Exercise rehabilitation**<br>((sarcopenia[Title] OR "skeletal muscle"[Title]) AND ("heart failure"[Title] OR "coronary artery disease"[Title]) AND (exercise[Title] OR training[Title] OR rehabilitation[Title])) AND english[Language] AND ("2021/01/01"[Date - Publication] : "2026/10/27"[Date - Publication])	35
Q4	**HFpEF muscle mechanisms**<br>("skeletal muscle"[Title] AND (HFpEF[Title] OR "heart failure with preserved ejection fraction"[Title]) AND (mitochondri*[Title/Abstract] OR inflam*[Title/Abstract] OR musclin[Title/Abstract] OR LOXL2[Title/Abstract] OR SIRT3[Title/Abstract] OR proteostasis[Title/Abstract])) AND english[Language] AND ("2021/01/01"[Date - Publication] : "2026/10/27"[Date - Publication])	21
Q5	**Nutrition and weight management**<br>((nutrition*[Title] OR "protein supplementation"[Title] OR "caloric restriction"[Title] OR "weight loss"[Title]) AND "heart failure"[Title] AND (sarcopenia[Title/Abstract] OR "muscle mass"[Title/Abstract] OR "muscle strength"[Title/Abstract] OR "body composition"[Title/Abstract])) AND english[Language] AND ("2021/01/01"[Date - Publication] : "2026/10/27"[Date - Publication])	34

Counts represent search returns rather than numbers of included studies. Records overlap across queries. The publication-date window applies to this revision-stage update, which supplemented the existing bibliography. Retrieval and relevance assessment were targeted and non-exhaustive.

Abbreviations: HFpEF, heart failure with preserved ejection fraction; PMID, PubMed identifier.

Supplementary Table S2

Table S2. Population eligibility, diagnostic basis, and applicability of selected rehabilitation studies to cardiovascular sarcopenia.

Study and population	Diagnostic basis at baseline	Applicability to cardiovascular sarcopenia	Assessment period and scope of inference
**CAD pilot** [68]<br>40 adults aged ≥60 years with coronary artery disease	Low BIA-derived muscle quantity and low handgrip strength were required.	Direct evidence in cardiovascular patients meeting an explicit mass-and-strength definition.	12-week intervention, with assessment 3 months after intervention. Short-term strength and performance benefits do not establish sustained diagnostic recovery.
**REHAB-HF functional sarcopenia analysis** [37]<br>343 participants classified; 169 with functional sarcopenia	SDOC criteria: low handgrip strength and slow gait speed. Muscle quantity was not measured.	Definition-specific direct evidence in cardiovascular functional sarcopenia.	Physical function at 3 months; clinical events at 6 months. Findings cannot establish recovery under definitions requiring low muscle quantity.
**REHAB-HF parent trial** [15]<br>349 older adults hospitalized with acute decompensated heart failure	Sarcopenia was not required for enrollment.	Indirect evidence for treating diagnostically defined cardiovascular sarcopenia; directly informs rehabilitation in a broader acute-heart-failure population.	12-week rehabilitation; physical function at 3 months and clinical events at 6 months. Performance improvement does not establish diagnostic recovery.
**Brubaker et al.** [14]<br>88 patients with stable obesity-related HFpEF	Sarcopenia was not required for enrollment.	Indirect evidence for cardiovascular sarcopenia; evaluates adding resistance training to caloric restriction and aerobic training.	20-week intervention and assessment; no post-intervention persistence assessment. The strength estimate favored added resistance training but remained imprecise; skeletal muscle mass declined in both groups.
**OptimEx-Clin** [48]<br>180 patients with stable HFpEF	Sarcopenia was not required for enrollment.	Indirect evidence for cardiovascular sarcopenia, principally concerning exercise capacity.	Assessments at 3 and 12 months. Peak oxygen uptake is distinct from muscle quantity, strength, and diagnostic sarcopenia status.

Study and population	Diagnostic basis at baseline	Applicability to cardiovascular sarcopenia	Assessment period and scope of inference
**SPRINTT** [85]<br>1519 community-dwelling adults aged ≥70 years	Trial-defined physical frailty and sarcopenia: low appendicular lean mass, SPPB 3–9, and ability to walk 400 m independently. Concomitant CVD was not required.	Indirect population evidence for cardiovascular sarcopenia.	Follow-up up to 36 months. The primary comparison involved 1205 participants with SPPB 3–7. Mobility findings do not directly estimate treatment effects in cardiovascular sarcopenia.

Applicability describes the relationship between the study population, diagnostic definition, and review question; it is not a formal certainty grade. Direct evidence in a diagnostically defined population does not itself demonstrate diagnostic recovery. Recovery requires reassessment of the required components of the same operational definition used at baseline.

The REHAB-HF parent trial [15] and functional sarcopenia analysis [37] are related reports from one randomized trial and should not be counted as independent replication. The 343 participants in the secondary analysis represent those with the baseline measurements required for classification; 169 met the SDOC functional criteria.

CAD, coronary artery disease; BIA, bioelectrical impedance analysis; CVD, cardiovascular disease; HFpEF, heart failure with preserved ejection fraction; SDOC, Sarcopenia Definitions and Outcomes Consortium; SPPB, Short Physical Performance Battery.

Reference numbers correspond to the bibliography of the accompanying revised manuscript.
